# On Mentagra and Tinea Capitis (Favus)

**Published:** 1890-02

**Authors:** 


### ON MENTAGRA AND TINEA CAPITIS (FAVUS).

By S. L.

The *Allgemeine Hom. Zeitung*, No. 19, 1889, contains a report of the meeting of the Saxon physicians, from which we extract the following:

*Villers* reports a partial failure in treating a heavily crusted mentagra, whose base was protruding and swollen. It appeared first on left side, and healed under the use of Kali-chrom. Then it came out on the right side, the hairs of the beard fell out, and now it failed to yield to Kali-bichr., Arsen., Silicea, or Thuja. Kali-iod. and Mezereum were spoken of. *Sybel* recommends Cod-liver oil to soften the crusts and Schüssler's Kalium-chloratum or Calcarea sulphurica. *Henze* uses Olium papaveris to soften the crusts, and relies on Hepar internally. He treated a boy of nine years with heavy crusts on the face where Calcarea ostr. failed ; a layman led his attention to Oleum terebinth$^3$, which cured the case. *Stifft* saw excellent effects in the Leipzig polyclinic in eruptions on the face with formation of crusts from Iod.-sulph.$^3$ in a boy of five years. *Heuser* reported an obstinate case of tinea capitis favosa maligna in an orphan girl of twelve years, who was afflicted with it since her third year. The mother of the girl had it for years and died from it. The child was delicate, anemic, the hair, cut short, was covered as with mold, matted, the whole scalp covered with thick crusts, showing cup-like cells. She was treated for three years in the orphan asylum without the least result, and the utmost care was unable to destroy the vermin. From the edges of the hairy scalp oozed a clear, gluey, foul-smelling fluid. Cervical glands hard, swollen, and painless. Her treatment so far consisted in the external use of Mercurial ointments and Cod-liver oil internally. Several children in that city had died from the same disease. Heuser ordered a few drops of Oleum anisi upon the head and covered with a cloth; on the following day Olive oil thickly laid on over the scalp, and after a few hours to be washed off with water and the yolk of an egg. The lice and the nits were killed and never showed themselves any more. Internally Graphite, Vinca minor, and Oleander were tried and failed, when she was put under constitutional treatment, Kali-iod.$^{1}$ three drops morning and evening. After two weeks a decided amelioration could be noticed, but also a severe Iodine coryza, which lasted a week. Sulphur$^{6}$ did not do much and therefore Heuser returned to Kali-iod.$^{3}$ for two weeks. More improvement and still more coryza, so that frequent omission of the drug was necessary, but in ten months she was cured and remained well. She is now married and has healthy children. Heuser acknowledges that now he would prefer the 30th potency as a constitutional remedy. *Villers* often uses Staphisagria for the lousy smell of a favus, and does not think it necessary to have the hair cut. *Henze* cured a moist eczema on the neck of a girl, with a foul discharge, with Arsen.$^{5}$, a dose morning and evening, without despoiling her of her hair. *Heuser* observed a case where disseminated papules at the edges of the hair changed to itching vesicles; by scratching these became confluent and covered by bright-red, bloody crusts. The child was very thirsty, drank often, but little at a time, felt weak and prostrated. Arsen. and Rhus failed, but Magnesia muriatica cured, which is also an antidote to Arsen. *Tschortner* cured an elderly gentleman suffering from tinea favosa by ordering a strict vegetarian diėt and Sulphur$^{30}$.

---

Villers' case of mentagra soon turned into a discussion of scrofulous skin-diseases, especially to tinea favosa, and we see them recommending clinically: Kali-chrom., Kali-bichrom., Kali-iod., Kali-mur., Magnesia-mur., Sulphur and Sulphur-iod., Arsen., Silicea, Thuja, Graphite, Oleand., Vinca minor, etc., the preparations of Kalis and of Iod. being the favorites. The skin trouble being an emanation not only of scrofulosis, but in one case even of hereditary psora, we felt rather astonished not to see antipsoric constitutional treatment put into the foreground, and Tschortner's recommendation of a strict vegetarian diet has our full approval. We know now that many a case of epilepsy, too often resting on a psoric diathesis, becomes incurable, because the little and the adult epileptic will regale himself with animal food, and thus may prostrate the action of a well-selected remedy. A mild, nourishing diet, an abstention from anything stimulating, is the *sine qua non* to a successful treatment of any skin-disease, and it would be well if the physicians of our present day would adhere more closely to the dietary management of their patients as laid down by Hahnemann in the *Organon* and in the first volume of *Chronic Diseases*. Regulation of diet first, and medicine becomes of secondary importance. Cleanliness inside and outside, on the external and internal skin, and the diseased poisons circulating in unhealthy blood will not find habitation in a pure body.

Heuser destroyed the whole brood of lice by a few drops of Oil of Anise, which reminded me of a remark made by a physician in the late French Congress, that the active principle, the poisonous one, in the beverage called Absynthe or Vermouth, is not Absynthe at all, but the Oil of Anise which is contained in that drink, and late old-school journals speak of Menthol in the same direction. Looking at our *Materia Medica* and at the *Cyclopedia of Pathogenesis*, we read nothing of that power to destroy vermin in either remedy, and still we know from toxicological experiments the influence of this kind of drugs on the nervous system.

We can easily see why the Kali salts are in these psoric affections favorites with our German colleagues, for it is well known that they all exert a deleterious, and hence also a curative effect over the manufacture of the blood, qualitatively as well as quantitatively—a weak heart is so characteristic of a Kali, where the other symptoms correspond. Such children, showing their stigma on their face, may apparently look plump, so that the old proverb was, the more eruption, the healthier the child, but we deal here with a false and deceiving plethora, and the muscular weakness—again a characteristic of the Kalis—will crop out, and still what a difference is between them. Thus we read how Heuser cured that obstinate case of malignant tinea with the steady use of the Iodide of Potassium, and if he only would have given it from the start in higher potencies these coryza aggravations might have been obviated.

The individuality of the patient suited the individuality of the drug, but its application was faulty. It is well known that Kali-iod. is a great remedy in tertiary syphilis, and may not scrofulosis be too often the punishment of the third and fourth generation for the sins of their ancestors? If it were not going too far, we might say there is nothing refined in the skin trouble and the remedy suits the disease, affecting more the lowest tissues of the body. What Kali-iod. is for distant emanation from syphilis, Kali-bichromicum is for the residue of a gonorrhœaic poison; and no wonder Villers tried it after the failure of such sycotic remedies as Thuja, Sarsaparilla, Silicea. While the Iodide caused a vesico-pustular eruption, the bichromate causes a vesicle with depressed centre, and just like small-pox, the pustule suppurates, and on healing leaves a cicatrix. Let us study one of Schüssler's favorite drugs, his Kali-muriaticum, his special remedy for fibrous exudations in any tissue, and in the treatment of cutaneous efflorescences which are, after all, exudations. It holds, therefore, with him a high place, especially in eczema, which has been developed after vaccination. But when Schüssler adds to it "with bad vaccine matter," he is only partially right, for we witnessed eczema, erysipelas, furunculosis, etc., after vaccination with perfectly pure vaccine, when the psoric poison needs only an impulse to show its presence by outward manifestations. Comparing Potassium Chloride with Thuja, we find them both indicated for bad effects of vaccination and of gonorrhœa. If Kali-iod. suits more torpid scrofulosis, Kali-brom. ought to be more often used in florid scrofulosis, but children suffering from the latter are only rarely affected with these disgusting eruptions, and still we read in the Pathogenesis of Bromium of pimples and boils, and practitioners used Bromium successfully in profusely discharging malignant scald-head with unbearable fetor and extreme tenderness of the scalp and for foul ulcers. The well-known acne of the Bromide of Potassium ought to lead more frequently our attention to this remedy, which on account of its abuse by the old school is too much neglected in our school. Just so the Iodide of Arsenic became a standard drug long ago, while the Brom-arsen. still awaits its proving and its more frequent application in disease, as especially in scrofulous skin-disease the waters containing Brom-arsen., or those in Ashe County, N. C., have earned a solid reputation. It would be worth while again to compare Arsenicum and its combinations with the different combination of Sulphur, for dryness of the skin and burning pains are characteristic of both of them. Thus we might go on comparing drugs, for there is no more delightful study than our *Materia Medica*, and find grains of gold where others throw them away as cumbersome trash.

What a useless task this classification of skin-diseases, for no two authorities agree, and it is well that for the relief of the patient the name is of very little importance. But in studying out with mathematical precision the drug which will be selected for its removal by its similarity to the state of the patient, let us not forget that something may be of greater importance than even the selection of the remedy. "Sublata causa tollit effectus" is no idle dream, and it is the duty of the conscientious physician to put his patient in such a sanitary state that his infinitesimal dose is not obstructed in its dynamic action by clogged material. Our German colleagues need no excuse for using also external means to cleanse the patient from his uncleanliness, and we cannot do better than follow rules which teach that cleanliness is next to Godliness.

